# Our Current Understanding of the Heterogeneity in Prostate Cancer and Renal Cell Carcinoma

**DOI:** 10.3390/jcm12041526

**Published:** 2023-02-15

**Authors:** Sha Zhu, Junru Chen, Hao Zeng

**Affiliations:** Department of Urology, Institute of Urology, West China Hospital, Sichuan University, Chengdu 610041, China

## Abstract

Urological cancer is a collective term for cancers of the bladder, kidney, ureter, penis, prostate, and testicles. Last year, more than 444,000 people were diagnosed with urinary cancers in the United States. In this review, we talk about the complexity of prostate and kidney cancer.

## 1. Prostate Cancer

Prostate cancer covers a rather long natural disease history. During this time, the nature of this disease undergoes drastic shifts. In most cases, patients are diagnosed with localized hormone-sensitive prostate cancer (HSPC), and some will eventually enter the lethal stage called metastatic castration-resistant prostate cancer (mCRPC). Besides the most common TNM classification, there are two other angles to clinically evaluate the disease: one according to the invasion of tumor cells (localized, locally advanced, and metastatic), and the other based on the sensitivity of tumors cells to androgen-deprivation therapy (ADT) (HSPC, CRPC). The combination of these two methods enables clinicians to understand the comprehensive aggressiveness and severity of a patient’s tumor.

Prostate cancer cells generally begin as hormone-sensitive, meaning their growth relies upon the presence of androgens, making ADT the cornerstone therapy. The initial responses to ADT are rather good; however, this is not long-lasting, and all patients become insensitive to the treatment sooner or later. On a cellular level, the treatment resistance denotes that these tumor cells have managed to bypass the dependence on androgens, either through androgen receptor (AR)-driven (intra-tumoral androgen synthesis or AR amplification) or AR-indifferent (neuroendocrine differentiation) mechanisms [1].

Currently, treatment for prostate cancer includes active surveillance, radical prostatectomy, radiotherapy, anti-androgen therapy, and some investigative therapies [2,3]. Especially for advanced prostate cancer, the choices for systemic treatments are vast, which target different malignant mechanisms in tumors, such as kinome deregulation, DNA damage and repair, and cell cycle abnormalities. Thus, understanding the heterogeneity within a big patient population is crucial in tailoring effective and personal treatment plans for patients.

It has been well established that prostate cancer is of a multifocal nature, i.e., multiple tumor foci that occur and develop independently, suggesting a polyclonal origin. This distinctive feature intrinsically denotes the significant heterogeneity of prostate cancer. Recent years have seen the emergence of many valuable descriptive sequencing studies that revealed prostate cancer’s complexity in different disease stages [4,5,6,7,8,9].

The mutational profiles differ considerably between localized and metastatic diseases. The Cancer Genome Atlas Research Network sequenced 333 primary prostate carcinomas and demonstrated that this cohort could be divided into seven subtypes featured by ETS fusions or SPOP, FOXA1, and IDH1 mutations. Furthermore, 26% of tumors are still included in neither of the subgroups, which reinforces that prostate cancer is indeed highly molecularly heterogeneous [5]. Significant heterogeneities in localized prostate cancer were also found at the cellular level using single-cell sequencing [10]. A multi-region sequencing study revealed that more genetically heterogeneous tumors harbored inherently worse disease outcomes [10]. The majority (over 90%) of mCRPC patients harbored clinically actionable mutations, but single nucleotide variants (SNVs) are rarely seen in localized diseases, which are instead dominated by non-SNV mutations [8,11]. DDR mutations are enriched in mCRPC patients, and twice that in localized patients [5,8]. Interestingly, localized tumors with BRCA2 mutations have a mutational profile similar to metastatic tumors rather than localized ones [12]. The importance of the BRCA2 mutation in early prostate cancer development has also been studied in another paper [13].

Besides disease stages, differences also exist among different pathological subtypes. According to the 2016 WHO classification of prostate tumors, there are at least twelve subtypes, including acinar adenocarcinoma, prostatic intraepithelial neoplasia, intraductal carcinoma, ductal adenocarcinoma, urothelial carcinoma, squamous neoplasms, basal cell carcinoma, neuroendocrine tumors, mesenchymal tumors, hematolymphoid tumors, miscellaneous tumors, metastatic tumors, tumors of the seminal vesicles [14]. Acinar adenocarcinoma is the most common pathological subtype, and some other subtypes have different prognostic indications. For instance, intraductal carcinoma of the prostate (IDC-P) and neuroendocrine prostate cancer (NEPC) strongly suggest unfavorable patient outcomes.

The name IDC-P describes tumor cells growing within the pre-existing ducts and acini of the prostate. Our group has focused on IDC-P for a decade. We discovered that IDC-P indicated a worse patient prognosis in almost each disease stage (localized disease, mHSPC, mCRPC) [15,16,17]. We also observed that IDC-P might be innately resistant to standard ADT [18] and that heterogeneity within IDC-P was associated with an altered efficacy of standard first-line therapies for patients with mCRPC [19]. Following our clinical observation, we initiated several sequencing-based studies to profile the characteristics of IDC-P. Our results from circulating tumor DNA (ctDNA) sequencing of patients with AC and IDC-P showed predominantly increased deleterious DNA repair alterations in IDC-P carriers [20]. Moreover, the frequent NCOR2 mutations in tumors with IDC-P indicated a distinct phenotype of AR pathway alteration. Following these findings, we then measured the homologous recombination deficiency (HRD) score, a result-oriented method to display the genomic instability status of tumors, in patients with or without IDC-P components in their tumors [21]. Our data suggest that IDC-P pathology represents higher HRD scores in prostate cancer, and tumors with IDC-P might have different driving mechanisms for high HRD scores than tumors without IDC-P. The HRD score was also prognostic in aggressive prostate cancers harboring IDC-P.

NEPC belongs to neuroendocrine tumors of the prostate and is a lethal stage that often occurs due to lineage plasticity after long-term ADT treatment. Tumors in this stage no longer depend on AR signaling; therefore, the expression of AR, PSA, and PSMA is generally down-regulated. Tumor suppressors RB1 and TP53 losses, activation of certain oncogenes, and some epigenetic changes are believed to be key players during this process [22]. Besides these two subtypes mentioned above, other pathological entities also show genomic characteristics, such as ductal adenocarcinoma, basal cell carcinoma, etc.

## 2. Renal Cell Carcinoma

Renal cell carcinoma (RCC) also represents a spectrum of heterogeneous diseases with different histological subtypes and distinct molecular alterations. Histologically, RCC consists of three major subtypes: clear cell RCC (ccRCC), papillary RCC (pRCC), and chromophobe RCC (chRCC). Despite a lower incidence, there are over ten other subtypes, such as collecting duct carcinoma, tubulocystic RCC, TFE3-translocation RCC, TCEB1-mutated RCC, fumarate hydratase–deficient RCC (FH-RCC), medullary carcinoma, and oncocytoma [23]. Among them, ccRCC is the most common form, accounting for approximately 75% of all RCC cases [24].

Different subtypes of RCC manifest distinct histological features [14]. Microscopically, ccRCC consists of tumor cells arranged in nests or tubules surrounded by a rich vascular network, with clear cytoplasm commonly filled with lipids and glycogen. Further, PRCCs often present as carcinomas with a prominent pseudo-capsule, composed of papillae formed by delicate fibrovascular cores. Traditionally, pRCCs are separated into type 1 pRCCs with a scanty cytoplasm and type 2 pRCCs with an abundant eosinophilic cytoplasm. Classic chRCC tumor cells show a prominent cell membrane, irregular nuclei, perinuclear halo, and a pale to eosinophilic cytoplasm. Several other rare RCC subtypes lack typical morphological features and often show a wide range of histological patterns, such as TFE3-translocation RCC and FH-RCC [25,26].

The metastatic patterns vary significantly between histological subtypes. Lung, adrenal, brain, and pancreatic metastases are more frequent in ccRCC; lymph node and peritoneal metastases are more frequent in pRCC; and liver metastases are more common in chRCC [27]. The clinical outcomes also differ according to the histology. Among the three major subtypes, chRCC has the most favorable prognosis [27]. While pRCC has a similar prognosis compared to ccRCC in a localized setting but worse clinical outcomes in a metastatic setting [28]. Patients with type 2 pRCC have significantly worse survival than those with type 1 pRCC [29]. Other rare histologies also exhibit more advanced TNM stages than ccRCC and are associated with higher mortality [30].

The molecular differences may be the basis of the heterogeneity in the clinicopathologic characteristics of different histological subtypes. Genetically, 3p loss is ubiquitously observed in ccRCC and has been reported to be the earliest event in ccRCC tumorigenesis [31]. VHL, SETD2, BAP1, and PBRM1, located in this region, are the most frequently mutated genes in ccRCC [29]. In addition, 5q gain and 14q loss, which include HIF1A, are also common in ccRCC. In contrast, pRCC is more heterogeneous and exhibits totally different genomic alterations compared to ccRCC. Type 1 pRCC is associated with the frequent gain of chromosomes 7 and 17, and the less frequent gain of chromosomes 2, 3, 12, 16, and 20 [32]. While in type 2 pRCC, only the loss of chromosome 22 occurs consistently as a specific copy number alteration [29]. For specific gene alterations, type 1 pRCC is characterized by MET alterations, whereas type 2 pRCC is associated with CDKN2A silencing and SETD2 mutations [32]. The loss of one copy of the entire chromosome has been frequently observed in chRCC [33]. Overall, chRCC shows apparently fewer somatic mutations than ccRCC and pRCC, with significant mutations of TP53 and PTEN [29]. Several other molecularly defined RCCs also have unique genomic alterations. FH-RCC, TFE3-translocation RCC, and TCEB1-mutated RCC are characterized by FH-inactivation alterations, TFE3-related fusions, and TCEB1 mutations, respectively [25,26,34].

Differences in RNA expression patterns also contribute to the heterogeneity of different RCC histological subtypes. Compared with pRCC and chRCC, ccRCC shows an elevated expression of angiogenesis, metabolic processes, and cell cycle signatures, which provides the molecular rationale for the use of anti-angiogenesis treatments in patients with advanced ccRCC [29]. The increased expression of amino acid metabolic processes and cilium signatures is unique in pRCC. Type 2 pRCC further exhibits a higher expression of the NRF2 antioxidant response pathway compared to type 1 pRCC [32]. Tumors could be destroyed by the immune cells in the microenvironment. Still, this immune response is often suppressed by the increased expression of immune checkpoint regulators in tumor cells and the corresponding inhibitory machinery. With the exception of Th17, IL-8, and CD56bright NK cell signatures, ccRCC shows nearly universal up-regulation of immune signatures compared to pRCC and chRCC, which partly explains the differential efficacy of immune checkpoint inhibitors in different RCC subtypes [29,35].

Heterogeneity exists even within one specific histological subtype. A multi-omics study revealed seven molecular subsets with distinct angiogenesis, immune, cell-cycle, metabolism, and stromal signatures in patients with advanced ccRCC from the IMmotion 151 trial [36]. Moreover, each subset had different genomic alteration profiles and varied responses to the atezolizumab+bevacizumab and sunitinib treatments. A recent meta-analysis enrolling 14,696 patients with ccRCC demonstrated that metastases possessed a significantly increased number of gene mutations and copy number alterations than primary tumors [37]. We previously performed whole-exome and RNA sequencing on 63 TFE3-translocation RCCs and found five molecular clusters with differential gene expression patterns, TFE3 fusion subtypes, copy number alteration profiles and survival outcomes [25].

In addition to inter-tumoral heterogeneity, intra-tumoral heterogeneity is also significant in understanding the tumor biology and evolutionary trajectory. A previous study from the UK performed whole-exome and RNA multi-region spatial sequencing on four patients with metastatic RCC and reported that over 60% of all somatic mutations were not detected across every tumor region [38]. In addition, gene-expression signatures of good and poor prognosis were detected in different regions of the same tumor. Similarly, the TRACERx Renal study analyzed primary tumors and metastases from 100 patients with ccRCC through multi-region genomic profiling [39,40]. This study showed variability in the extent of intra-tumoral genomic heterogeneity between tumors and that patterns of intra-tumoral genomic heterogeneity were associated with tumor evolution, progression and survival outcomes.
